# PathwayFinder enables linked exploration of cell types and gene modules in spatially resolved transcriptomics data

**DOI:** 10.1016/j.isci.2026.118043

**Published:** 2026-10-29

**Authors:** Chang Li, Julian Thijssen, Thomas Kroes, Lisa Koorneef, Thomas Höllt, Louise van der Weerd, Boudewijn Lelieveldt

**Affiliations:** 1 Department of Radiology, Leiden University Medical Center, Leiden, the Netherlands; 2 Department of Anatomy and Embryology, Leiden University Medical Center, Leiden, the Netherlands; 3 Computer Graphics and Visualization, INSY, TU Delft, Delft, the Netherlands; 4 Department of Human Genetics, Leiden University Medical Center, Leiden, the Netherlands; 5 Delft Bioinformatics Lab, INSY, TU Delft, Delft, the Netherlands; 6 Department of Informatics, University of Bergen, Bergen, Norway

**Keywords:** data visualization, visual analytics, spatial transcriptomics, bioinformatics

## Abstract

The rapid development of spatially resolved transcriptomics technologies has provided opportunities and challenges for understanding biological systems. Interpreting these data requires integrated analysis of gene expression and cell types within their spatial context, however, existing tools fall short in scaling this linked interactive exploration to large-scale datasets. We present PathwayFinder, a visual analytics application designed to bridge the gap between gene-centric and cell-centric analysis. Unlike standard workflows that view cells and genes in isolation, PathwayFinder links them through a multi-scale interactive interface. This allows researchers to explore the relationships between transcriptomic profiles and tissue architecture, for example, by selecting a cell type to instantly discover its potential marker genes, or brushing gene modules to identify cell populations expressing them and their spatial distributions. Through this tight integration of gene, cell, and spatial views, PathwayFinder facilitates rapid discovery of co-expressed gene modules, specialized cell subpopulations, and functional hypotheses in spatial contexts.

## Highlights

• PathwayFinder enables linked exploration across cell, gene module and spatial view
• Multi-scale navigation reveals cell subpopulations and candidate marker genes
• Integrated Gene Ontology enrichment supports functional discovery
• Immediate visual feedback supports exploratory hypothesis generation

## Graphical abstract

**Figure:**
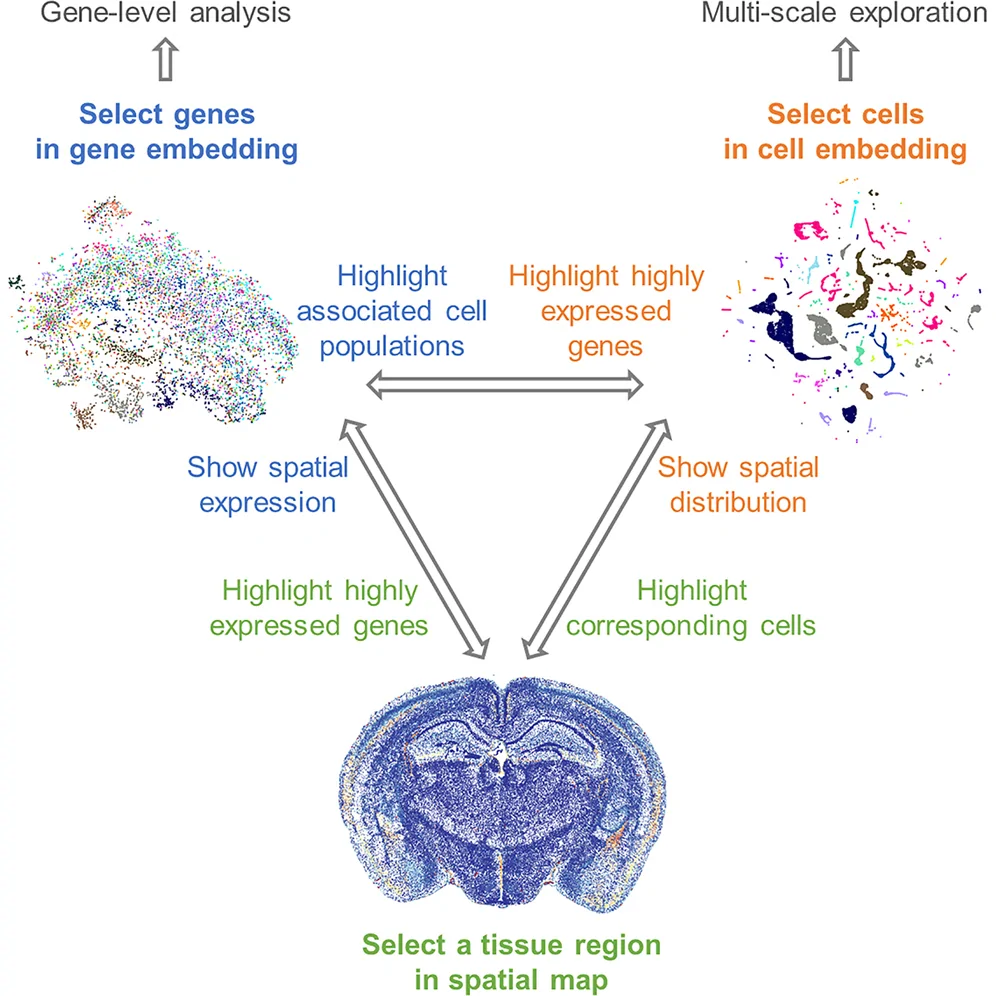

## Teaser

Computational bioinformatics; Systems biology; Transcriptomics

## Introduction

Large-scale spatially resolved transcriptomics (SRT) data resources are rapidly emerging, capturing gene expression across millions of cells along with their spatial coordinates (in 2D and 3D). These datasets offer opportunities to explore cellular heterogeneity, tissue organization, and diverse biological functions, but also present significant challenges for analysis and visualization due to their complexity and scale.^1^

Data visualization techniques, particularly dimensionality reduction methods, such as t-distributed Stochastic Neighbor Embedding (t-SNE)^2^ or Uniform Manifold Approximation and Projection (UMAP)^3^ are commonly used to explore and analyze these large-scale datasets. By projecting the high-dimensional expression data to low-dimensional space while preserving high-dimensional similarities, the cell embedding can reveal the underlying data structure, such as transcriptomic heterogeneity and expression gradients.^1^^,^^4^^,^^5^ To enhance scalability for increasingly large datasets, hierarchical dimensionality reduction methods such as hierarchical stochastic neighbor embedding (HSNE)^6^ have been developed to analyze data at different levels of resolution.^7^

Similarly to cell embeddings, dimensionality reduction methods can be applied on the transposed cell-by-gene expression matrix, to infer gene-gene similarities and identify gene modules that co-express across cells and cell types. To biologically interpret gene modules, enrichment analysis tools such as g:Profiler^8^ can be used to identify statistically significant Gene Ontology (GO) terms^9^ associated with a gene set. Enrichment analysis confirms the potential functional coherence of co-expression modules and it can also provide the full gene list linked to specific GO terms, facilitating further exploration.

Moreover, it has been shown that transcriptomic cell types often appear at specific spatial locations within tissues.^10^ Incorporating spatial context with these embeddings further helps in understanding the transcriptomic heterogeneity, gene similarities and anatomical information. Current downstream analysis of SRT data^11^^,^^12^^,^^13^ relies heavily on Python or R packages to perform the above introduced tasks such as dimensionality reduction, clustering, differential gene expression etc. While powerful, these script-based approaches require coding expertise and treat individual analytical tasks in isolation, limiting the flexibility of data exploration. Therefore, interactive visualization platforms^14^^,^^15^^,^^16^^,^^17^^,^^18^ have been developed to facilitate data inspection via cell embeddings or spatial maps. However, these tools still lack the deeper downstream facilities, and focusing on only one axis of the data (cells, genes, or spatial tissue organization) may hide valuable insights from the other axes.

To address this, BrainScope^19^ introduced linked maps for samples, genes, and brain regions, providing an integrative visualization of the transcriptional landscape. By selecting points in the gene map, researchers can identify co-expression modules; by selecting points in the sample map, they can examine anatomical similarities in gene expression. However, the dual-embedding strategy in BrainScope was designed for small numbers (<150) of bulk transcriptomics samples and is not scalable to datasets with millions of cells.

To address these challenges, we present PathwayFinder, a multi-scale visual analytics application that extends the dual-embedding concept to large SRT datasets, with integrated spatial context. To achieve such integrated multi-scale exploration, we adapted WAOW-Vis,^20^ which links together two hierarchical embeddings based on the strength of their relationships. In our case, this means clusters in the cell embedding will show strong links to genes that are highly expressed within them, enabling rapid identification of candidate marker genes. And vice versa, gene modules in the gene embedding will show strong links to cell populations they are highly expressed in, revealing potential cell-type specificity. We further enhanced this with a spatial view visualizing where selected cells are located in the tissue, along with its 2D or 3D spatial context and facilities such as on-the-fly GO term enrichment to further inspect gene modules. Unlike conventional scripting-based analyses, which often require extensive coding and rely on static plots, PathwayFinder offers immediate, interactive visual feedback by the tight integration of all these views with any user interaction. In the following sections, we first introduce the system design and interactive facilities, and then demonstrate its utility with use cases that illustrate how this workflow supports functional discovery and hypothesis generation.

## Results

### System overview

PathwayFinder is designed for linked exploration of three domains (cells, genes, and spatial context) in large-scale SRT data. Users can begin from any of these domains and iteratively explore marker genes and co-expressed modules, gain insights into distinct molecular profiles, and uncover spatial co-expression or anatomical patterns. By allowing observations in one domain to guide subsequent exploration in the others, PathwayFinder supports exploratory hypothesis generation with immediate visual feedback.

A screenshot of the interface is shown in Figure 1B. The system accepts input in the form of a cell-by-gene expression matrix, along with their corresponding spatial coordinates and optionally cell-type metadata. The pipeline begins by computing a multi-scale cell embedding using HSNE^6^ based on the cell-by-gene matrix, which captures the dataset’s global cellular structure at the top level and enables users to “drill down” and inspect cell subpopulations for detailed analysis. A gene embedding is generated by applying t-SNE^2^ to the transposed cell-by-gene matrix, where genes with similar expression profiles across cells are placed in proximity. To visualize the relationships between these entities, we implement a node-link representation. 1D t-SNE embeddings are generated for both cells and genes, which serve as “nodes”. The “links” are drawn between cells and genes when expression levels exceed a user-defined threshold, visualizing the strongest gene-cell connections (details in Methods section). Cell embeddings are colored by user-selected cell type metadata, and gene embeddings are color-coded to match the dominant cell type where they show maximal expression. By highlighting cells or displaying expression levels in the spatial map, we further connect expression patterns of cell clusters to anatomical regions. A metadata panel displays detailed information about the user’s current selection. For cell selections, the panel displays cell type proportions and lists genes with high Log2 fold change (Log2FC) relative to the full dataset. For gene selections, it displays the gene list and their expression distribution across cell types. In order to retrieve a potential functional profile of a gene set, users can initiate an on-the-fly GO term analysis via a button press. The retrieved GO terms will be listed as a ranked table in the metadata panel, and GO term associated genes can be highlighted in the gene embedding.

**Figure 1:**
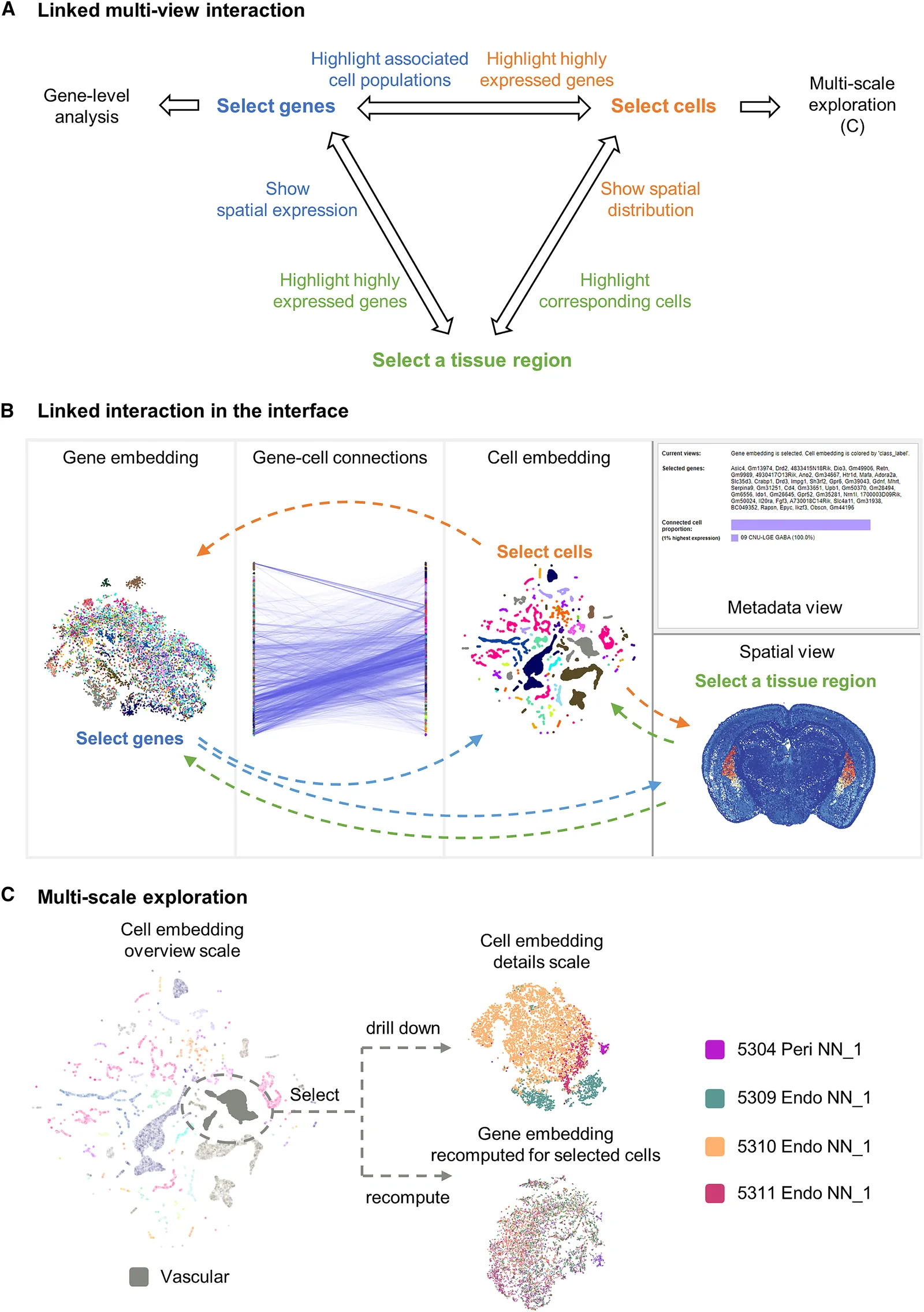
Overview of the PathwayFinder system (A) Illustration of the linked multi-view interaction among the cell embedding, gene embedding and spatial map. Exploration can start from any of the three views, with selections propagated to the others. Interactions starting from the gene embedding are shown in blue, those starting from the cell embedding in orange, and those starting from the spatial map in green; the same color scheme is used in (B). Subsequent gene-level analysis can be explored beyond the interaction of the three main views. Cell selections can additionally trigger multi-scale drill-down, as detailed in (C). (B) Screenshot of the interactive interface containing a gene t-SNE embedding colored by dominant cell type, gene-cell connections, a cell HSNE embedding colored by cell-type annotation, a metadata view, and a spatial map colored by gene expression level. Colored arrows illustrate interactive linking among these views: selecting genes in the gene embedding (blue) updates the cell embedding and spatial map, selecting cells in the cell embedding (orange) updates the other two views, and selecting tissue regions in the spatial map (green) updates the other two. (C) Illustration of the multi-scale exploration workflow. From a global view of cell embedding, a region of interest in the cell embedding can be drilled down to get a detailed view, and the gene embedding is recalculated based on the selected cell subset.

PathwayFinder emphasizes interactive exploration by allowing users to brush data items in a spatial, gene or cell centric view and observe corresponding updates in the other views in real time (Video S1). These interactions are synchronized across all views in real-time, as illustrated by the interaction diagram in Figure 1A and the colored arrows in Figure 1B. Selecting genes in the gene embedding proportionally resizes the cells in the cell embedding based on the selection’s average expression, aiding in the discovery of potential co-expressed modules or marker genes. Conversely, selecting a cell cluster resizes genes in the gene embedding based on the Log2FC of the selected population relative to the entire dataset. This visual encoding immediately highlights top-expressed genes, supporting the interpretation of distinct molecular profiles. In the meantime, the spatial map updates to show cell locations of selected cells or average gene expression intensity of selected genes, uncovering anatomical patterns or spatial co-expression. Furthermore, users can select cells of interest on the spatial map to examine the corresponding cell types and identify genes that are highly expressed within these spatially defined regions. These interactive explorations allow observation, domain knowledge and subsequent questions to guide analytical steps, supporting the generation and refinement of biological hypotheses.

Video S1. Demonstration of interactive exploration with PathwayFinder using MERFISH mouse brain ABCA-4 from Zhang et al^21^

To handle the large quantity of cells in large-scale datasets, we choose the HSNE^6^ algorithm to create a multi-scale view of cell embeddings. In this multi-scale iterative workflow (Figure 1C), biologists can start with a global perspective and “drill down” by refining selections in the cell embedding. Upon zooming in to a subpopulation, the system allows for the recalculation of the gene embedding specific to that selection. This iterative approach ensures that subtle biological signals, such as rare cell types or context-dependent marker genes, can be identified and compared against the global overview.

### PathwayFinder enables interactive exploration across genes, cells, and spatial views

To demonstrate the biological utility of PathwayFinder, we applied it to three publicly available datasets to illustrate two distinct exploratory workflows: starting from a cell-centric or a gene-centric query. We used publicly available datasets from the Allen Brain Cell Atlas (ABC Atlas), comprises a coronal section of the multiplexed error-robust fluorescence *in situ* hybridization (MERFISH) mouse brain from Yao et al.^1^ and sagittal sections from Zhang et al.^21^ as well as a Xenium Prime mouse brain hemisphere dataset.^22^

To validate the system’s ability to identify cell-type specific genes, we first applied PathwayFinder to the coronal section 38 from Yao et al.^1^ The cell embedding reflects the similarities between cells, providing an overview of the cell type landscape. As shown in Figure 2, selecting distinct visual clusters in the cell embedding reveals the highly expressed genes and at the same time highlights the spatial locations of selected cells. In many cases, we observed that genes highlighted by size in the gene embedding share colors with the selected cell types, confirming the validity of color-coding genes by their top-expressing cell type. This color-coding aids users in guiding their initial exploration for gene modules intuitively. Furthermore, clusters of highly expressed genes associated with specific cell types often form visually distinct groups within the gene embedding, suggesting cell-type specificity and shared functional roles. The linked spatial map further supports this interpretation by integrating cell type information to anatomical structures.

**Figure 2:**
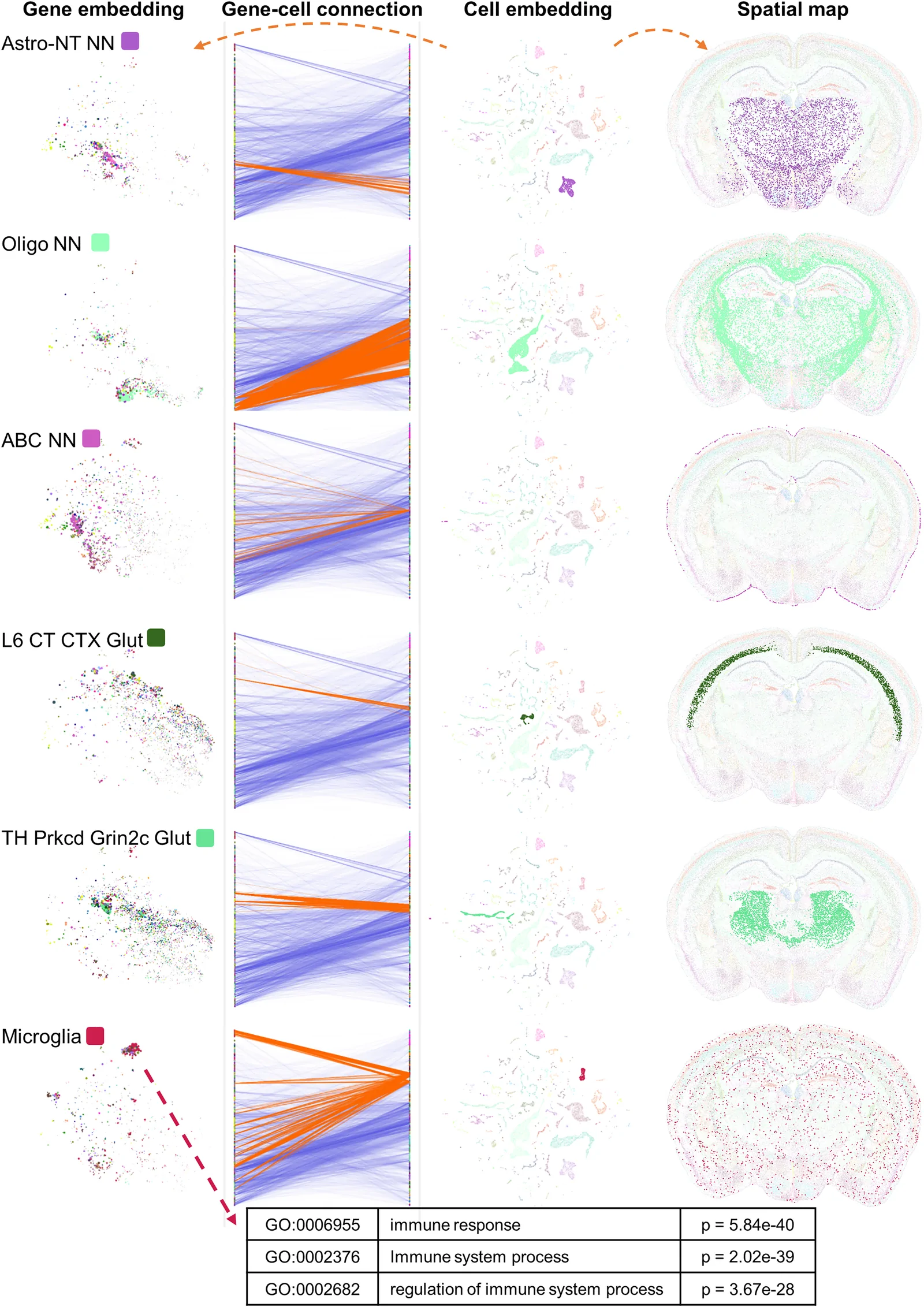
Selecting clusters from the cell embedding highlights highly expressed genes and their spatial locations Columns from left to right contain: gene embeddings colored by the top cell type (point size proportional to Log2FC of selected cells relative to the entire dataset); gene-cell connections; cell embeddings colored by cell-type annotations with selected cells highlighted; spatial maps colored by cell-type annotations with corresponding cells highlighted. For microglia, enrichment analysis of the associated gene cluster identified three top GO terms, all pointing to immune functions. All *p* values are Bonferroni corrected. This figure uses data from coronal section 38 from Yao et al.^1^ Astro-NT NN, non-telencephalon astrocyte, non-neuronal; oligo NN, oligodendrocyte, non-neuronal; ABC NN, arachnoid barrier cell, non-neuronal; L6 CT CTX Glut, layer 6 corticothalamic-projecting glutamatergic cortical neuron; TH Prkcd Grin2c Glut, thalamus *Prkcd/Grin2c* glutamatergic neuron.

The connections between the cell embedding and gene embedding visually indicate the strength and distribution of associations between cell types and gene clusters. For example, Figure 2 demonstrates that selecting the Oligo NN (oligodendrocyte, non-neuronal) cluster reveals a primary gene cluster highly expressed within this cell type in the gene embedding, while highlighting a strong, major gene-cell connection. In contrast, selecting the microglia NN cluster identifies one distinct primary gene cluster and several additional highly expressed genes scattered outside this primary cluster, with a more branched pattern of gene-cell connections. This visual contrast reflects differences in transcriptional specificity, as oligodendrocytes show a high specificity and microglia show a combination of unique markers and broadly active genes shared with other populations.

Similarities in gene expression may point to shared cell type identities and biological functions. Clusters observed in the gene embedding typically represent functionally related genes. Besides exploring gene expression via selected cell types, users can also select gene clusters directly to inspect their expression across different cell types and their spatial expression patterns. To demonstrate the discovery of gene modules, we applied PathwayFinder to MERFISH ABCA-4 with three sagittal mouse brain sections from Zhang et al.^21^ Figure 3 shows the functional characterization and spatial expression patterns for the 4,000 genes with the highest density values in the gene embedding. The GO enrichment analysis results confirm that co-expression within these clusters relates to distinct biological pathway and cellular functions, validating the system’s capability for real-time and flexible linkage of cell-type-specific expression patterns to biological functions.

**Figure 3:**
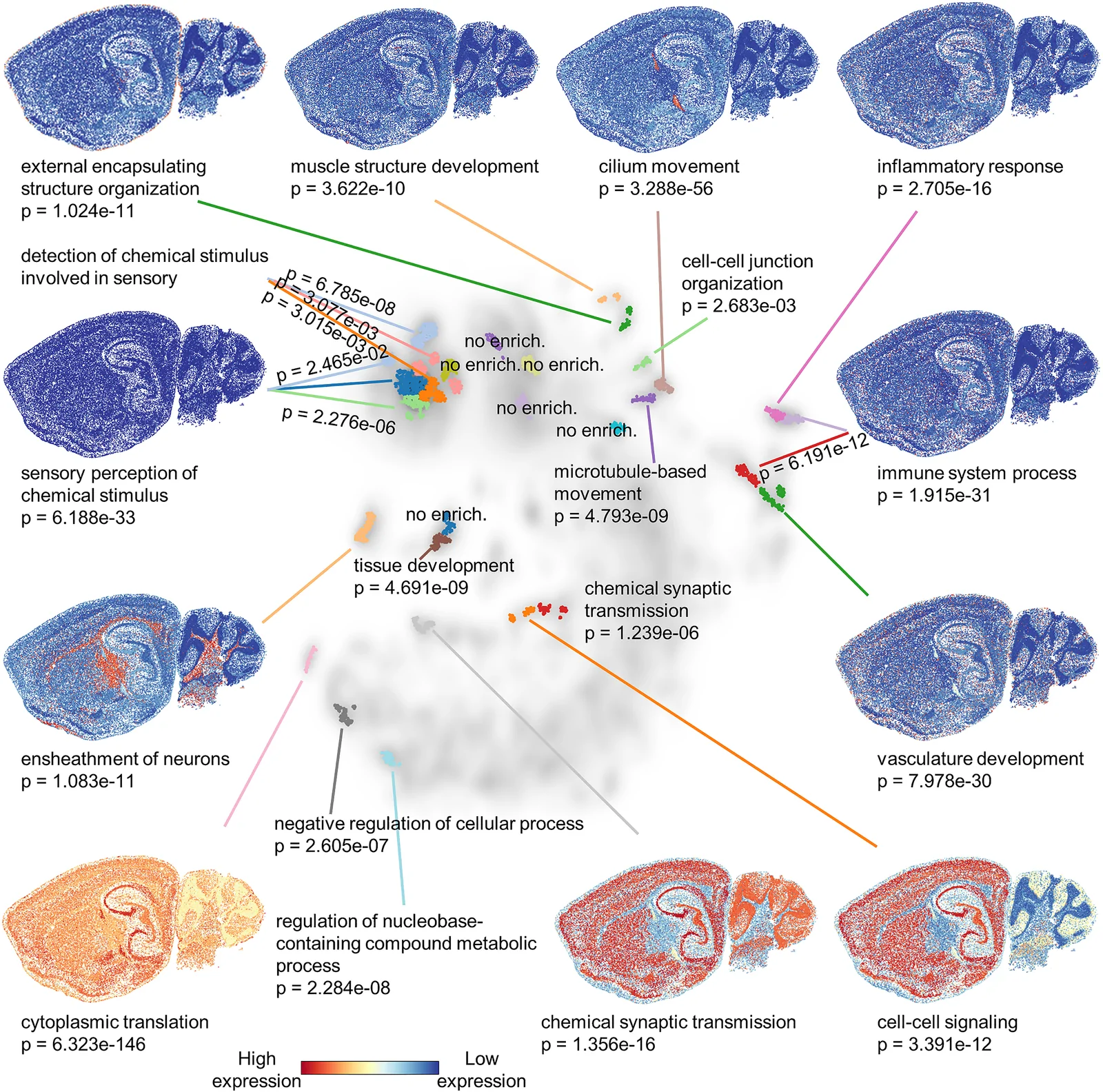
Functional characterization of the gene embedding 4,000 genes with the highest densities in the gene embedding are clustered. The biggest 30 gene clusters were characterized using g:Profiler. Only the most significant GO::biological process term is presented for each cluster, and spatial maps colored by the average expression of clusters are also provided (red indicates high expression, blue indicates low expression). All *p* values are Bonferroni corrected. The gene modules are provided in Table S2. This figure uses data from ABCA-4 from Zhang et al.^21^ with all three sagittal slices included in computation, the third sagittal slice is shown here.

To further demonstrate the general applicability on different SRT protocols, we applied PathwayFinder to a 10× Xenium Prime mouse brain hemisphere dataset,^22^ to demonstrate the exploration on datasets without cell type annotations. In Figure 4A, selecting a cell population directly from the t-SNE cell embedding highlights the corresponding cells on the spatial map, revealing their anatomical locations. Genes with Log2FC larger than 2 are on-the-fly identified and sent for enrichment analysis, providing immediate functional interpretation of the chosen cell population. To independently validate the biological identity of the selected cell populations, we employed MapMyCells (RRID: SCR_024672) afterward to generate annotations for this originally unannotated dataset. It’s important to note that these annotations were not used in our exploratory analysis; instead, selected populations are labeled according to the most dominant cell type assigned by MapMyCells only for confirmation. Figure 4B shows exploration from the gene embedding, where interactively selecting a gene cluster highlights the associated cell population and its enriched GO terms. Figures 4A1/4B2 (immune response) and 4A3/4B4 (striatum development) show two examples of how PathwayFinder provides an interactive, two-way visual feedback between gene modules and cell populations, enabling a rapid biological interpretation in unannotated datasets.

**Figure 4:**
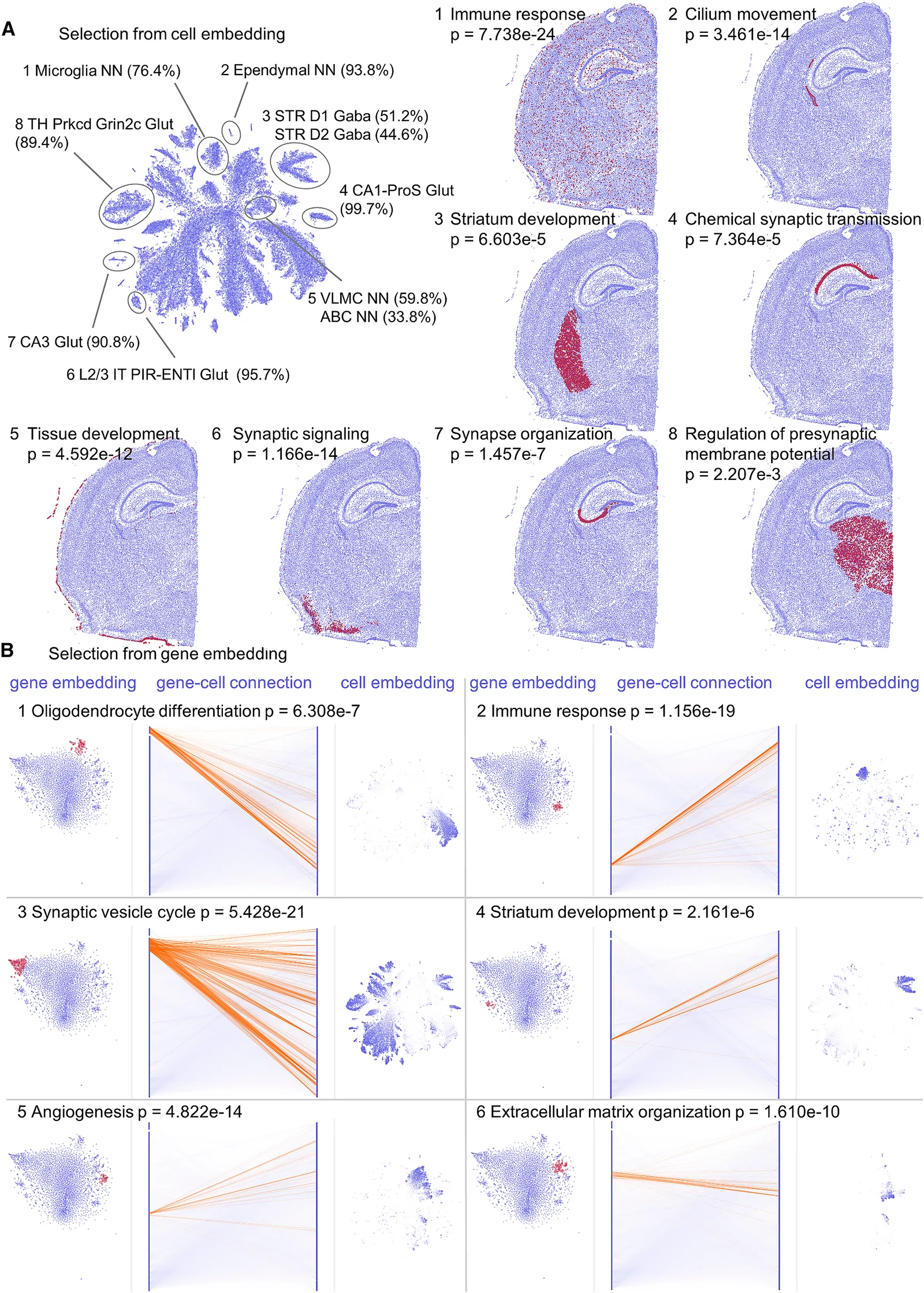
Exploration of Xenium Prime mouse brain hemisphere dataset demonstrates PathwayFinder’s applicability on different protocols and without annotations (A) Selection of a cell population from the t-SNE cell embedding highlights its anatomical location on the spatial map. Genes with Log2FC larger than 2 are identified and sent for GO enrichment, revealing functions of the selected cell population. Reference cell types were assigned via MapMyCells (RRID:SCR_024672) afterward to confirm the relevance of the retrieved GO terms; the selected population is labeled with the dominant cell type and its proportion within the selection. These MapMyCells annotations were not used during the exploratory analysis. (B) Selection of a gene cluster in the gene embedding highlights the corresponding cell population. The retrieved GO terms reveal functional characterization of the selected genes. Together with (A), the two-way interaction between cell and gene embeddings enables an immediate visual feedback and functional interpretation even in unannotated datasets. The gene modules are provided in Table S3. Genes were characterized using g:Profiler, with all 5006 genes in this dataset sent along as background gene set. Only the most significant GO::biological process term is presented in the figure, and all *p* values are Bonferroni corrected. Microglia NN, microglia cell, non-neuronal; ependymal NN, ependymal cell, non-neuronal; STR D1 Gaba, striatum D1 medium spiny neuron GABAergic neuron; STR D2 Gaba, striatum D2 medium spiny neuron GABAergic neuron; CA1-ProS Glut, CA1-prosubiculum glutamatergic neuron; VLMC NN, vascular leptomeningeal cell, non-neuronal; ABC NN, arachnoid barrier cell, non-neuronal; L2/3 IT PIR-ENTl Glut, layer 2/3 intratelencephalic-projecting piriform-lateral entorhinal glutamatergic neuron; CA3 Glut, CA3 glutamatergic neuron; TH Prkcd Grin2c Glut, thalamus *Prkcd/Grin2c* glutamatergic neuron.

We then compared the gene embedding from Xenium Prime mouse brain hemisphere dataset^22^ (5,006 genes) with MERFISH mouse brain coronal section 38^1^ (8,460 imputed genes), to evaluate the consistency of gene embeddings across datasets. These two datasets share 2,565 common genes. Selecting gene clusters in one dataset and highlighting them in the other revealed a general correspondence between gene co-expression modules (represented as local neighborhoods in the gene embedding), indicating that functional gene modules are preserved across datasets (Figures S1A and S1B). For example, immune-related and oligodendrocyte-related genes showed consistent patterns across the two datasets. Some gene clusters, such as those highly expressed in the thalamus, showed weaker correspondence. To investigate this further, we inspected the genes that were not in the same neighborhood between the datasets, and found that each dataset showed different spatial expression patterns due to data-specific or protocol-dependent differences (Figure S1C). Specifically, these differences likely arise from three major technical aspects: (1) the comparison of computationally imputed transcriptomics from scRNA-seq data (MERFISH) against directly measured spatial transcripts (Xenium); (2) inherent platform differences in probe design and optical decoding sensitivities; and (3) biological variance driven by not perfectly matched sectioning locations and individual animal differences. Nevertheless, for the majority of gene clusters, PathwayFinder captures preserved and data-specific transcriptional structures across datasets.

In addition to interactive selections from the cell and gene embeddings, users can select anatomical regions directly from the spatial map or from the annotations. This enables rapid identification of the cell types within a region and the genes showing region-specific expression.

Together, these usage scenarios demonstrate the utility of PathwayFinder. The system allows users to interactively explore gene, cell, and spatial views and offers immediate visual feedback, providing more flexibility than pre-defined standard analysis pipelines. These results confirm that PathwayFinder reproduces known biological insights and enables hypothesis generation in both annotated and unannotated datasets.

### Multi-scale analysis of 3D SRT data of the hypothalamus reveals fine-grained details of transcriptional similarities in neuroendocrine regulation

Our system supports a multi-scale workflow, which enables the exploration of large-scale data. Following “Overview first, details on demand”, users can easily navigate between broader overviews and detailed, selection-specific analyses. This hierarchical approach is essential for analyzing complex tissues, enabling the inspection of small cell populations and subtle transcriptional variances.

Here, we demonstrate the biological utility of our multi-scale exploration approach using the hypothalamus subset of the Yao et al. MERFISH mouse brain^1^ (Figure 5A). For this example, we worked with a biologist interested in corticotropin-releasing hormone (*Crh*). *Crh* can act as a neuropeptide, affecting anxiety, autonomic outflow, and other (stress) responses, or as a neurohormone that regulates the secretion of stress glucocorticoid hormones. These different roles can be distinguished by the location of *Crh* expression and its co-expressed cell-type markers. We first searched for and selected *Crh* in the gene embedding. Upon selection, the linked cell embedding and spatial view automatically update to reflect *Crh* expression level (Figure S2). The spatial view confirms *Crh* expression in specific hypothalamic regions, including the paraventricular nucleus (PVN), where both neuroendocrine and preautonomic CRH neurons reside (Figure 5C; Figure S2). In the meantime, the overview-level cell embedding highlights its strong expression in hypothalamus GABAergic (HY GABA) and hypothalamus glutamatergic (HY Glut) cell classes in the whole hypothalamus, indicating that *Crh* is involved in both inhibitory and excitatory processes (Figure S2). The linked multi-view system enables to subsequently select cells in the cell embedding that are only present in the PVN, which appears to be the HY Glut population. To go more into detail, we subsequently selected the HY Glut population in the cell embedding, and colored the newly computed cell embedding by the more refined cell type annotation “cluster” in the ABC Atlas taxonomy (Figure 5B; Figure S2). At the detailed level, we observe two distinct *Crh*-expressing neuronal populations (Figure 5D): *Crh* neurons co-expressing *Scgn*, classifying them as neuroendocrine neurons; and *Crh* co-expressing *Reln*, classifying them as the preautonomic neurons, as previously established in the recent scRNASeq PVN atlas.^23^

**Figure 5:**
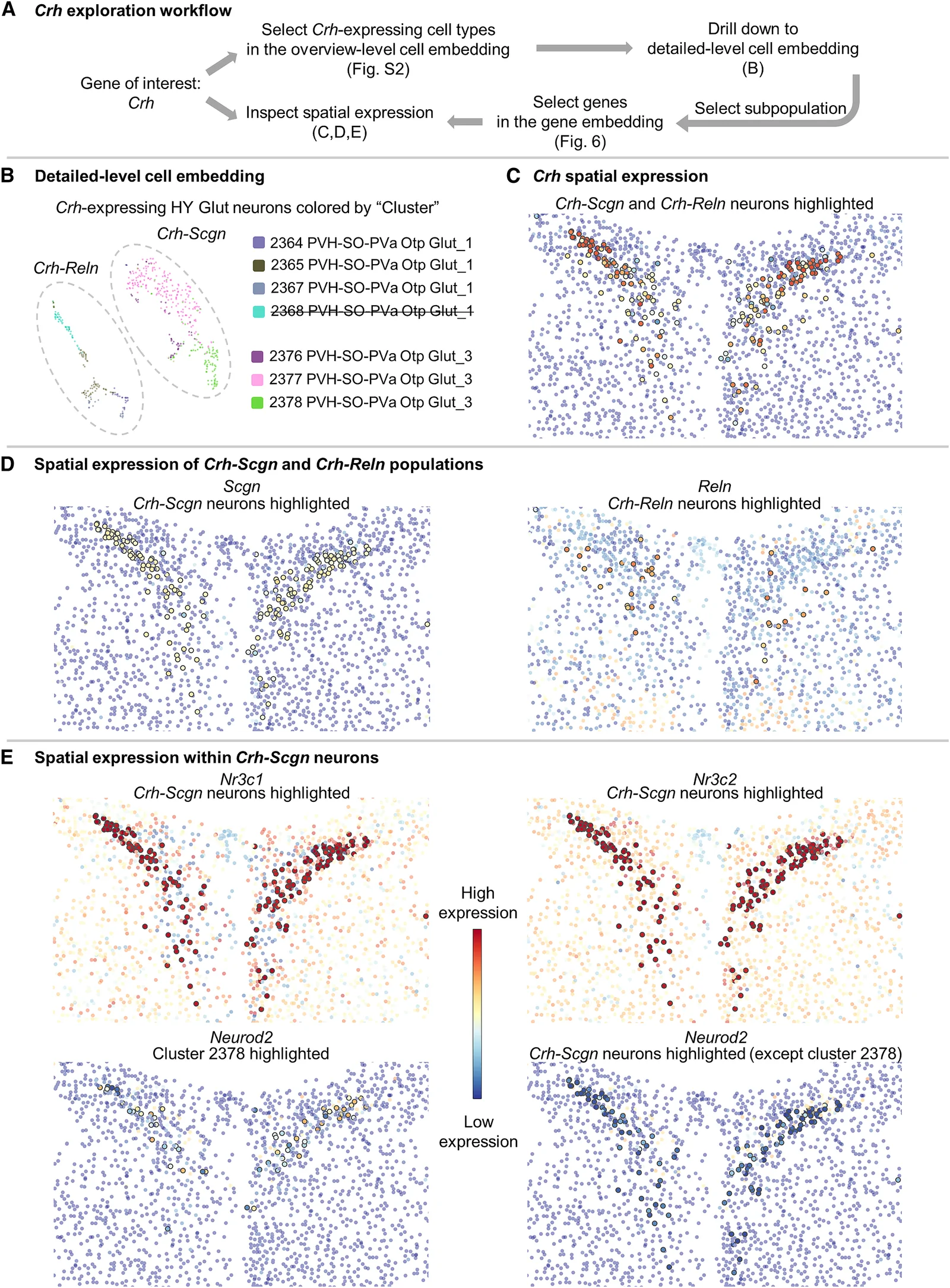
Multi-scale exploration results of Crh-expressing populations (A) Example exploration workflow starting from a gene of interest, in this case *Crh*. (B) A detailed level of cell embedding, colored by “cluster” annotations. The embedding was drilled down from the selected *Crh*-expressing hypothalamus glutamatergic (HY Glut) neurons in the PVN at the overview scale. Cluster 2368 was excluded when highlighting cells in the spatial view due to its low *Crh* expression. (C) Spatial expression of *Crh* zoomed in the paraventricular nucleus (PVN) of coronal section 43. Expression levels are shown using a red-to-blue colormap, where red indicates high expression, blue indicates low expression. Highlighted cells are outlined with dark gray circles, the unhighlighted cells are set to 50% opacity for visual clarity. (D) Spatial expression of *Scgn* and *Reln* reveals two distinct neuronal populations. (E) Spatial expression of *Nr3c1*, *Nr3c2*, and *Neurod2* reveals potential subpopulation-specific responsivity to stress glucocorticoid hormones within *Crh-Scgn* neurons.

Furthermore, we discovered that the neuroendocrine *Crh*-*Scgn* population could be further subdivided into three subpopulations (annotated as cluster 2,376, 2,377 and 2,378 in the ABC Atlas taxonomy). All subpopulations express the two receptors for stress glucocorticoid hormones: the glucocorticoid receptor (GR, *Nr3c1*) and the mineralocorticoid receptor (MR, *Nr3c2*). In neuroendocrine *Crh-Scgn*, the GR is well known to mediate negative feedback on the stress HPA-axis. Although GR and MR can bind to the same DNA regulatory sequences to control gene expression, they also occupy distinct genomic sites, a difference partly determined by the coregulator *Neurod2*.^24^ While all neuroendocrine CRH subpopulations expressed GR and MR, only cluster 2378 co-expressed *Neurod2*, suggesting differential glucocorticoid responsiveness between the subpopulations (Figure 5E). The above workflow demonstrates that PathwayFinder serves as a visual exploration tool for rapid and interactive identification of potential markers for subpopulations, as well as for predicting subpopulation-specific responsivity. Definitive conclusions regarding the biological relevance of these findings, however, require subsequent statistical validation and/or wet-lab experiments.

Compared with existing approaches, the niche of PathwayFinder is that it provides an interactive and iterative exploration through selecting cell populations or genes, with immediate visual feedback across linked views. Rather than requiring users to formulate a specific question in advance and execute corresponding analysis scripts, PathwayFinder allows observations made during exploration to guide subsequent selections and enables users to combine visual patterns with domain knowledge and formulate hypotheses. For example, subpopulations can be selected interactively and visualized in multiple views simultaneously, allowing to verify both their spatial locations and their positions within the cell embedding. In the meantime, the gene embedding is dynamically updated to highlight genes with high expression in these populations. As demonstrated in Figure 2, the point size of each gene is rescaled proportional to its differential expression between the selected population and the whole dataset. This size-based highlighting enables the interactive selection of top genes that are specifically enriched in the selected *Crh-Scgn* neurons (Figure 6A). In parallel, their spatial expression patterns across the hypothalamus can be visualized in real-time, providing insight into the regional selectivity of the gene expression profile (Figure 6B). This can enable the identification of cell type markers (such as *Crh*, *Scgn*, and *Reln*); as well as other enriched genes that may generate new hypotheses regarding (functional) differences among the various *Crh* subclusters (such as *Neurod2*), thereby guiding future investigations. It is important to note that we used the annotations from the ABC Atlas taxonomy in this use case for metadata coloring in the visual interface, but the drill-down computation does not use these annotations, i.e., this multi-scale exploration pipeline can work without annotations.

**Figure 6:**
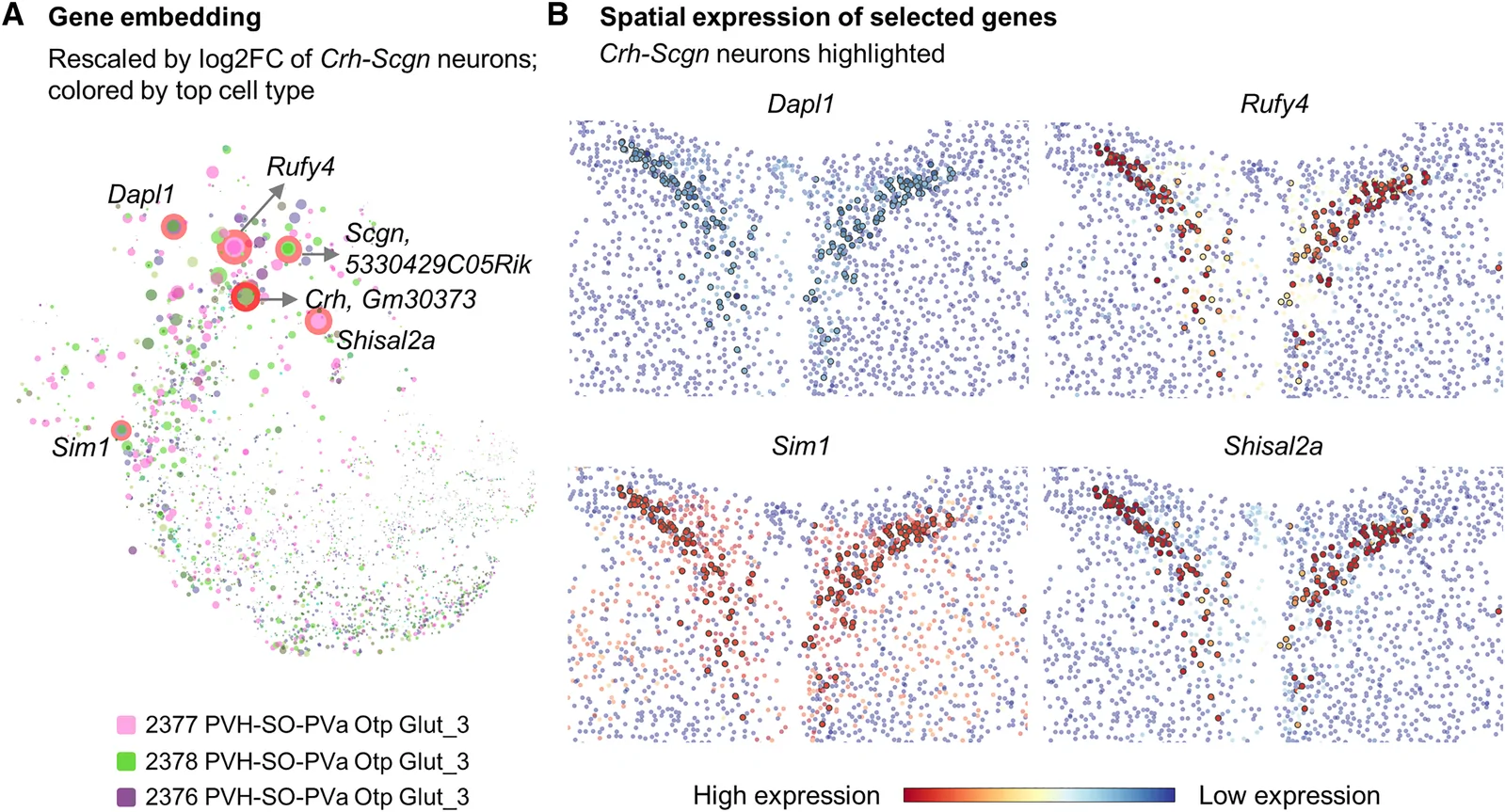
Selecting genes from the gene embedding interactively reveals diverse spatial expression of genes highly expressed in the selected *Crh-Scgn* neurons (A) Gene embedding in which point size is rescaled based on the Log2FC of *Crh-Scgn* neurons relative to the entire hypothalamus dataset. Each gene is colored in the same color as the dominant cell type. (B) Spatial expression of genes selected in (A), with *Crh-scgn* neurons highlighted. Expression levels are shown using a red-to-blue colormap, where red indicates high expression, blue indicates low expression. Highlighted cells are outlined with dark gray circles, the unhighlighted cells are set to 50% opacity for visual clarity.

In addition to the 2D view of the spatial maps, PathwayFinder also provides a complementary 3D view for 3D datasets (Figure S3). Users can isolate specific 3D structures of interest by selecting cells within the 2D spatial scatterplot or by clicking on existing anatomical annotations. Through linked selections, isolating a 3D structure updates the linked gene embedding and other views, enabling rapid identification of potential markers of the focused region.

### Iterative exploration followed by GO term retrieval reveals the link between gene expression and cell type composition

To complement the interactive views, PathwayFinder integrates on-the-fly GO enrichment analysis, providing immediate functional annotations of visual observations and facilitates exploratory hypothesis generation (Figure 7A). Although GO enrichment can also be performed using conventional analysis packages, PathwayFinder incorporates this step directly into the interactive exploration workflow. This feature allows users to conveniently connect the current cell, gene, and spatial context directly with functional biology. The GO term enrichment can be triggered at any stage during analysis. For instance, selecting a visual cluster in the gene embedding enables identification of common functions among genes with similar expression patterns. Alternatively, selecting specific cell types from the cell embedding or anatomical regions from the spatial map provides immediate functional annotations for genes highly expressed in these subsets.

**Figure 7:**
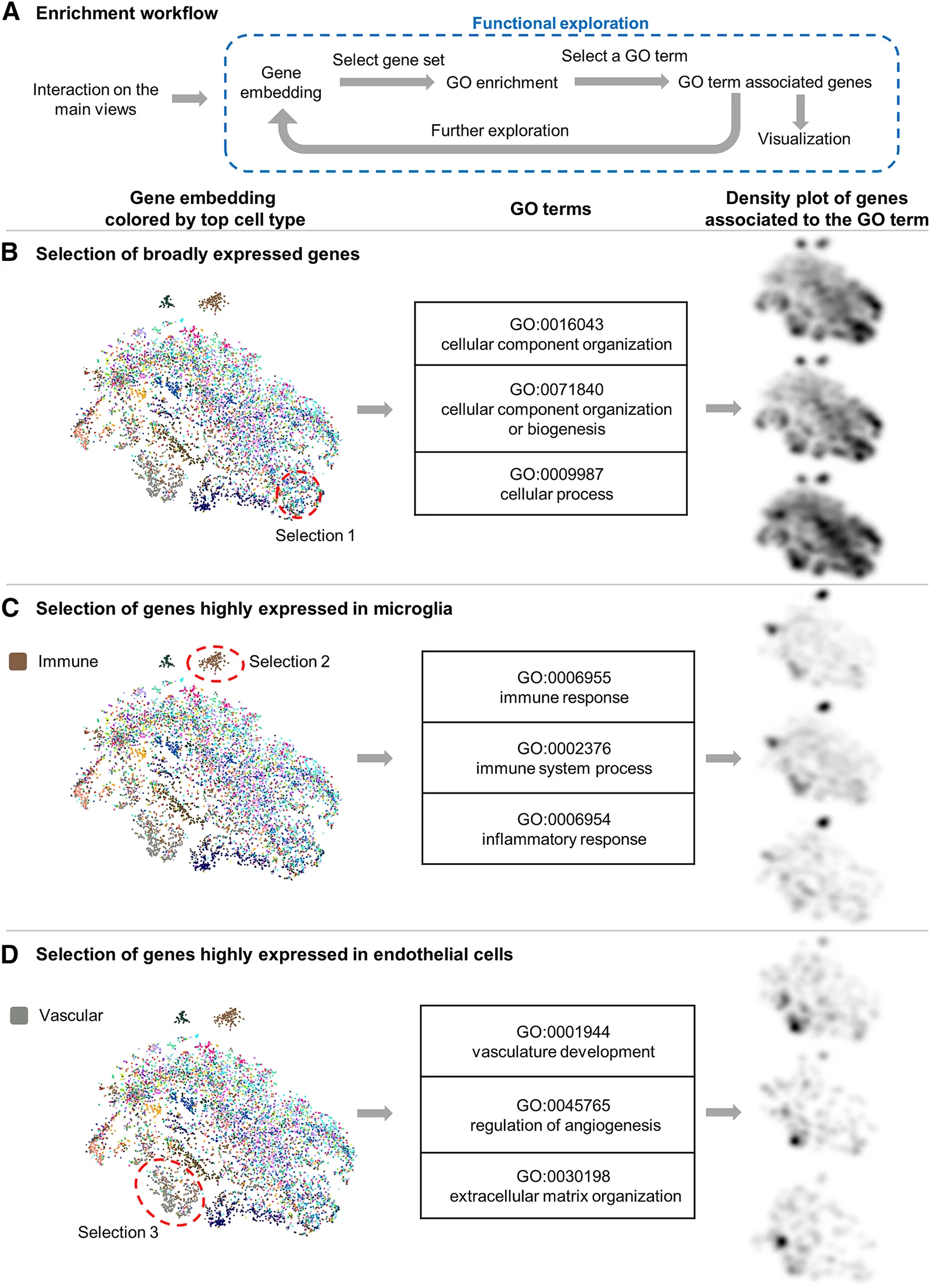
Density visualization of gene embedding reveals functional diversity across cell types and gene clusters (A) Exploration and enrichment workflow. Users interactively explore data with the linked three views, selected gene sets can be sent for on-the-fly GO enrichment. Associated genes of a selected GO term are visualized as highlighted on the gene embedding or as a density plot on the gene embedding, and then return to the main views for further exploration. (B) Housekeeping genes selected on the gene embedding (selection 1) show broad expression across most of the cell types. GO enrichment retrieves the general terms, with associated genes distributed broadly throughout the gene embedding. (C) A gene cluster highly expressed on microglia cells (selection 2) retrieves immune-related GO terms. The associated GO term “immune process” and “immune system process” reveals two distinct density hotspots in the gene embedding: the selected cluster and an additional immune-related gene cluster. The associated GO term “inflammatory response” highlights one strong hotspot, corresponding to the selected gene cluster. (D) Selecting endothelial cell clusters (Endo NN) reveals a corresponding gene cluster enriched for vascular-related GO terms (selection 3). The GO term “vasculature development” shows two distinct density hotspots: the selected one corresponding to Endo NN, and another corresponding to Pericyte NN and VLMC NN populations, confirming shared functions across vascular cell types. Selecting genes highly expressed in the VLMC cluster shows that they are also associated with the GO term “extracellular matrix organization”, with a single hotspot specific to genes highly expressed in the VLMC cluster.

Statistically significant GO terms are displayed in the metadata view. Clicking on a specific GO term highlights associated genes within the gene embedding. Users can then plot these highlighted genes into a separate scatterplot view by simple drag-and-drop, to visually assess the distribution and clustering patterns of the genes associated to the GO term. The scatterplot view also supports a density plot mode, which shows the gene hotspots on the GO term genes’ embedding coordinates, reflecting the coherent biological functions or broadly expressed patterns. This visualization can then be used to subdivide a GO term into smaller, high-density gene clusters.

To demonstrate the usage of PathwayFinder linking gene expression, cell types and functional annotation, we explored distinct scenarios with the GO term enrichment. First, selecting a group of broadly expressed genes (e.g., *Gapdh*) in the gene embedding revealed widely spread expression across cell types in the cell embedding. Interestingly, expression was notably lower in highly specialized subpopulations, such as vascular cells and microglia. GO term enrichment analysis retrieved the general term “cellular process”, with the associated genes distributed broadly through the whole gene embedding, confirming the expected general cellular functions of housekeeping genes (Figure 7B).

Second, selecting a cell cluster with annotation of microglia NN highlights a gene cluster with high expression in these microglia neurons. The retrieved GO terms of this gene cluster confirms the immune-related functions such as “immune response” and “immune system process”. Clicking on a GO term and plotting the associated genes in the density plot shows two distinct hotspots in the gene embedding, one is the initially selected gene cluster and another contains additional immune markers, such as *Cd7*, *Cd28*, and *Cd226*. The GO term “inflammatory response” highlighted a single strong hotspot in gene embedding, confirming the specificity of the initially selected microglia-expressing gene cluster (Figure 7C).

Third, selecting endothelial clusters (Endo NN) on the cell embedding highlights a gene cluster with corresponding color in the gene embedding, indicating endothelial-specific expression. GO term enrichment analysis retrieved “vasculature development” as a significant functional annotation. Further inspection of this GO term revealed multiple gene hotspots corresponding to distinct but related cell populations, such as Peri NN and VLMC NN. This finding highlights functional gene sets shared across multiple vascular-related cell types, illustrating cellular complexity and differentiation within the vasculature. Additionally, selecting genes highly expressed in the Endo cluster also retrieved the GO term “regulation of angiogenesis” whose associated genes formed a single strong hotspot in the gene embedding, indicating the role of endothelial cells in forming new blood vessels.^25^ Moreover, selecting genes highly expressed in the VLMC cluster retrieves the GO term “extracellular matrix organization”, with its associated genes forming a single strong hotspot, consistent with its specialized role in structural support (Figure 7D).

## Discussion

The rapid development of SRT technologies has produced increasingly large datasets, leading to new challenges for analysis and biological interpretation. Integrated exploration of gene expression and cell types in their spatial tissue context can reveal distinct cell populations, interactions and co-expression modules. However, existing tools fall short in interactivity, and in scaling to these evolving large-scale (2D or 3D) datasets.

Here, we presented a visual analytics application PathwayFinder for interactive exploration of SRT data by linking cell, gene and spatial views in a multi-scale manner. One of the main contributions of our system is its iterative and interactive workflow that links cell types, gene modules, and spatial expression, with integrated GO term enrichment analysis. Our system enables users to explore large and complex datasets intuitively. The real-time linked visual feedback from the multiple views helps the interpretability of biological findings and enables fast hypothesis generation.

In this manuscript, we qualitatively evaluated PathwayFinder using publicly available datasets across different protocols, demonstrating its capability to identify cellular diversity, cell-type-specific gene modules, and their functional roles. We then illustrated how our multi-scale exploration workflow identifies known marker genes and generates hypotheses through a use case starting from *Crh*. Furthermore, we showcased the utility of the integrated GO term analysis functionality, highlighting its value in revealing biological relationships between gene expression patterns and cellular compositions.

While computational programming packages^11^^,^^12^^,^^13^ offer comprehensive analytical pipelines, the script-based nature introduces friction during exploratory analysis. Iteratively redefining subpopulations, mapping them to spatial coordinates, and calculating marker genes for a specific population requires repeated script execution and switching between separate analysis and visualization steps. In contrast, PathwayFinder provides interactive, real-time facilities to support rapid visual discovery and exploratory hypothesis generation. Users can continuously refine cell populations, inspect associated genes, and visualize spatial patterns without rerunning analysis scripts. This shortens the process cycle between observing a pattern, formulating a hypothesis and visual examination. Furthermore, compared to existing interactive visualization frameworks^14^^,^^15^^,^^16^^,^^17^^,^^18^ such as Vitessce^16^ and napari-spatialdata,^18^ which provides integrative facilities to explore spatial data, PathwayFinder offers distinct visual analytics novelties. Specifically, we utilized the dual-embedding architecture that links the cell and gene dimensionality reduction spaces via biologically thresholded node-link plots. This linked representation allows users to directly examine the relationships between cells and genes and then visualize how they relate to spatial regions. By presenting these relationships with an interactive workflow, PathwayFinder can help users identify associations that may be difficult or time-consuming to identify through separate plots and analyses, thereby supporting the generation of new biological hypotheses. Compared to BrainScope,^19^ which visualizes sample-level data instead of cell-level embeddings, our approach adapted a hierarchical exploration pipeline to support scalable, multi-scale exploration. We also extended the GO term analysis to show all genes associated to a specific GO term and link the GO term to the density plot visualization in the gene embedding, enabling integrated functional interpretation of gene clusters. In comparison to WAOW-Vis,^20^ we extended the node-link visualization for SRT data, introducing color-coding and user-defined Log2FC threshold to assist identification of cell-type specific markers. In addition, we implemented additional interaction facilities with more flexible settings, providing more generalizability across diverse datasets.

Importantly, PathwayFinder is implemented as a desktop application composed of a set of plugins within the ManiVault Studio framework.^26^ This modular architecture enables flexible integration with other built-in plugins, allowing users to perform additional analysis with minimum efforts within the same environment, such as differential expression analysis or custom annotation. Furthermore, this framework enables researchers to easily save, share, and restore visual analytics states, supporting reproducibility and collaborative analysis.

While our demonstration mostly focused on large-scale SRT datasets with annotations, PathwayFinder is broadly applicable beyond these examples. First, the system remains functional if the data do not contain spatial coordinates, for instance scRNA-seq data. In this case, the cell and gene embeddings still allow interactive exploration of cell-gene relationships and functional annotation of gene modules. Second, PathwayFinder supports data without cell-type annotations, the embeddings can be explored without color coding (Figure 4), and annotations are intended for enhancing interpretability. Users may optionally apply external annotation transfer methods to add annotations from reference datasets, which is beyond the scope of this work. It can also be used to aid manual annotation of unlabeled datasets, where GO terms can help guide cell type labeling. Third, users are not restricted to the built-in HSNE and t-SNE implementations for dimensionality reduction, externally computed embeddings, such as UMAP, can be directly imported for the linked exploration. Fourth, the system is compatible with datasets of varying sizes. Smaller datasets are supported, while larger datasets naturally provide richer patterns in the embeddings. The gene embedding performs the best when a large number of genes are present, allowing to form distinct visual clusters of genes. Taken together, PathwayFinder provides general applicability and flexibility across datasets.

Overall, PathwayFinder offers an interactive visual interface for exploring large-scale SRT data. By dynamically updating linked views of gene, cell embedding and spatial map based on user interaction, PathwayFinder helps study the co-expressed gene modules, specialized cell subpopulations, and functional hypotheses.

### Limitations of the study

Our system supports hierarchical exploration of large-scale SRT data, however, its scalability is currently restricted by hardware memory constraints. Unlike programmatic pipelines that can process data sequentially in chunks, the full expression matrix needs to be loaded into memory to ensure the real-time interaction, which limits performance on datasets larger than the available hardware memory (details in Methods/Implementation and performance section). To address this and extend practical hardware limits, we offer the possibility to load the data in different precision, for example float32 and bfloat16. Also, we aim to extend the idea of binary representations of transcriptomic data in future versions of the tool. Another promising future direction is integrating on-disk storage and chunked data access, which could further reduce memory consumption. These developments would enhance scalability, making it feasible to support much larger datasets interactively.

Another limitation lies not in the system itself, but in the inherent sensitivity limitation of SRT data. To address this, PathwayFinder supports the import of imputed datasets. For example, the MERFISH datasets used in the case studies^1^^,^^21^ include gene expression imputed from scRNA-seq data, expanding the targeted spatial gene panel. In addition, integrating SRT data with other high-resolution datasets through frameworks such as KanCell^27^ may further enable whole-transcriptome exploration.

Finally, while our system provides a multi-scale exploration framework, there is significant room to expand these capabilities. Transitions across scales could be improved by implementing tracking facilities, enabling users to record their analytical history and hierarchical findings. Future development will also focus on enhanced 3D support, such as direct spatial brushing in the 3D space, as well as more advanced visual encodings that integrate statistical hypothesis testing directly into the exploratory views.

## Resource availability

### Lead contact

Further information and requests for resources and reagents should be directed to and will be fulfilled by the lead contact, Boudewijn Lelieveldt (b.p.f.lelieveldt@lumc.nl).

### Materials availability

This study did not generate new unique reagents.

### Data and code availability

• This paper analyzes existing, publicly available data. Links for the datasets are listed in the key resources table.
• The software installer, project files containing the data and GUI configurations, and source code are available at https://doi.org/10.5281/zenodo.22917815.
• Any additional information required to reanalyze the data reported in this paper is available from the lead contact upon request.

## Acknowledgments

This work received financial support from NWO Gravitation project BRAINSCAPES: A Roadmap from Neurogenetics to Neurobiology (10.13039/501100003246NWO: 024.004.012).

## Author contributions

C.L., L.v.d.W., and B.L. conceptualized the project. C.L., J.T., and T.K. implemented the software. C.L., L.K., and B.L. performed the analyses and interpreted the data. T.H. and B.L. supervised the project. C.L. and B.L. wrote the manuscript with assistance and approval from all authors.

## Declaration of interests

The authors declare that they have no competing interests.

## STAR★Methods

### Key resources table

**Table:** 

REAGENT or RESOURCE	SOURCE	IDENTIFIER
**Deposited data**		

MERFISH mouse brain imputed dataset	Yao et al.^1^	https://allen-brain-cell-atlas.s3.us-west-2.amazonaws.com/index.html#expression_matrices/MERFISH-C57BL6J-638850-imputed/20240831/
MERFISH mouse brain imputed ABCA-4	Zhang et al.^21^	https://allen-brain-cell-atlas.s3.us-west-2.amazonaws.com/index.html#expression_matrices/Zhuang-ABCA-4/20230830/
Xenium Prime mouse brain hemisphere dataset	10× Genomics^22^	https://www.10xgenomics.com/datasets/xenium-prime-fresh-frozen-mouse-brain

**Software and algorithms**		

PathwayFinder	This paper	https://doi.org/10.5281/zenodo.22917815
g:Profiler	Kolberg et al.^8^	https://biit.cs.ut.ee/gprofiler/gost
MapMyCells	Allen Institute	RRID: SCR_024672https://portal.brain-map.org/atlases-and-data/bkp/mapmycells

### Experimental model and study participant details

This study does not involve an experimental model or subject in the life sciences.

### Method details

#### Data

The input data is a cell-by-gene expression matrix, with spatial coordinates and optionally cell type metadata connected to each cell. PathwayFinder currently supports loading datasets in the h5ad and h5 file formats.

In this paper, we used existing, publicly available datasets to demonstrate the usage of the application. The imputed Yao et al. MERFISH data^1^ contains ∼4 million cells and 8,460 genes imputed from scRNA-seq data. We used section 38 and a hypothalamus subset in our analyses. The imputed ABCA-4 MERFISH data from Zhang et al.^21^ contains ∼220k cells and ∼30k genes imputed from scRNA-seq data. We took the ∼160k cells that passed the cell classification confidence score threshold and 20k highly variable genes, and applied a log2(x+1) transformation to the data. The Xenium Prime mouse brain hemisphere dataset^22^ contains 63,173 cells and 5,006 genes. The sizes of the utilized datasets are shown in Table S1.

#### Implementation and performance

PathwayFinder is implemented in C++ as plugins of the ManiVault plugin system^26^ for visual analytics application building. It can be downloaded, installed, and run as a standalone application.

PathwayFinder is designed for exploring datasets across varying scales. Computational resource requirements depend primarily on the data size and memory usage is the most significant factor. The exact memory usage and parameters for each dataset demonstrated in this paper are recorded in Table S1. To demonstrate scalability and performance, we tested the performance of all the datasets used in the analysis on two computers: a workstation with a 6-core, 3.80 GHz Intel processor, 128 GB of RAM and an NVIDIA Quadro 2200 GPU, and a laptop with a 6-core, 2.60 GHz Intel processor, 16 GB of RAM and an NVIDIA GTX 1660Ti GPU. Video S1 was recorded on the workstation, demonstrating a fully interactive experience. On the laptop, all pre-saved projects can be loaded with a short delay, and remain fully interactive. Furthermore, datasets exceeding physical RAM can still be loaded by utilizing operating system virtual memory (e.g., Windows drive paging), at the cost of longer loading times, while real-time interaction performance remains mostly unaffected once loaded. This demonstrates that PathwayFinder remains usable on typical computer hardware.

#### Dimensionality reduction

Dimensionality reduction is performed separately for cells and genes using the gene expression matrix and its transpose, respectively. The implementation is built upon t-SNE and HSNE plugins from the ManiVault framework,^26^ which provide customizable parameter settings as those available in HDILib.^6^^,^^28^

Users can choose between t-SNE or HSNE based on dataset size. When HSNE is selected, the workflow enables multi-scale exploration. Because the HSNE algorithm^6^ traverses a global k-nearest neighbor graph to drill down to a local subset, the cell-cell similarities are robustly preserved across different levels of scale. An initial overview embedding represents the global structure; selecting landmark cells triggers the computation of a detailed t-SNE embedding for the selected cell subset and its associated gene embedding. Users can visualize results and navigate between hierarchical levels (overview vs. detailed) by dragging and dropping the generated embeddings into the respective views.

Corresponding 1D embeddings are automatically generated upon the completion of the 2D embeddings. The 1D t-SNE embeddings are computed based on the 2D coordinates and optimized to align with the 2D y axis during gradient descent, while preserving the clustering structure present in the 2D embedding.

All dimension reduction parameters used in the analyses are listed in Table S1.

#### Cell-gene node link plot

From a visual design perspective, visualizing the massive number of connections between cells and genes presents significant challenges regarding visual clutter and overplotting. We employed a node-link plot representation because it preserves the geometric topology of the embeddings and allows the visual shape of connections to convey transcriptional specificity. In this layout, the nodes represent the 1D embeddings and the links illustrate the relationships between cells and genes based on their expression patterns.

To reduce overplotting in large datasets, our system uses a highlighting mechanism driven by brushing and linking. When no selection is made, blue lines connect cells and genes based on global expression levels, rendered at a low opacity to provide background context. A link is established between gene g and cell c if the expression value *E*_*g*,*c*_ exceeds a user-defined threshold relative to the data range:

(Equation 1) $$E_{g,c}>E_{g,\min}+T_{e}\;\left(E_{g,\max}-E_{g,\min}\right)$$

where *E*_*g*,*c*_ represents the expression of gene g in cell c, *E*_*g*,*min*_ and *E*_*g*,*max*_ are the minimum and maximum expression for the gene, and *T*_*e*_ ∈[0,1] is the threshold that can be interactively defined by the user through a slider.

When a selection is made via interactive brushing, the highlighted links corresponding to this selection are rendered in orange on the top visual layer. If genes are selected, links connect them to cells that satisfy the expression threshold described above.

Conversely, when cells are selected, links are connected based on differential expression between the selected cells and the whole dataset. In this case, a link is established between a selected cell subset *S*_*c*_ and a gene *g* if the log2FC exceeds a user-defined threshold:

(Equation 2) $$\max\left(0,\log\;2\left(\frac{\left(mean\left(E_{g,S_{c}}\right)+0.05\right)}{\left(mean\left(E_{g,All}\right)+0.05\right)}\right)\right)\;>T_{f}$$

where $mean\left(E_{g,S_{c}}\right)$ is the average expression of gene *g* within the selected cell subset *S*_*c*_, *mean*(*E*_*g*,*All*_) is the average expression of gene *g* within the whole dataset, *T*_*f*_ is the threshold that can be interactively defined by the user through a slider, the small offset 0.05 is to avoid the effect of very small overall expression.

#### Interactive linked views

The interface includes five linked components: a gene embedding, a gene-cell node-link plot, a cell embedding, a spatial map and a metadata view (Figure 1B, Video S1). Users can select data within the gene, cell and spatial views using rectangle, lasso, brush or polygon tools.

When genes are selected, point radii in the cell embedding are scaled to the mean expression of the selected genes. The spatial map is colored by the mean expression of the selected genes. In the node-link plot, edges connecting the selected genes are highlighted in orange. The metadata view lists the selected gene symbols (allowing for GO term enrichment via the ‘Enrich’ function) and displays a stacked bar chart showing cell type proportions for the top 1% of cells with the highest mean expression of the selected genes.

Conversely, selecting cells (in the cell embedding or spatial view) triggers a differential comparison against the whole dataset. The gene view scales point radii based on the normalized log2FC values. The node-link plot highlights connections between the selected cells and related genes in orange, while the metadata view updates to display associated gene symbols and selected cell type proportions.

#### GO term enrichment

GO term enrichment is performed on-the-fly using the g:Profiler^8^ API with a significance threshold of 0.05. This analysis is triggered whenever a gene set is defined, either through direct selection in the gene embedding or by identifying highly expressed genes derived from cell selection. Users can configure the target organism and significant correction method in the settings. To ensure statistical accuracy, background gene selection adapts to the data type: for datasets with at least 20,000 genes, all measured (or imputed) genes serve as the background set; for datasets with no less than 20,000, no specific background list is applied. The results are displayed as a ranked list of GO Biological Process (GO:BP) terms with associated *p*-values. Selecting a GO term highlights all associated genes present in the dataset (extending beyond the initial input list) within the gene embedding and stores them as a distinct subset for further inspection.

#### Density plot and clustering for gene embedding

To identify reproducible gene clusters within the gene embedding for Figure 3, we first computed a kernel density estimate (KDE) over the 2D coordinates of 20,000 highly variable genes using a Gaussian kernel from Scikit-learn.^29^ The optimal KDE bandwidth was selected via 5-fold cross-validated grid search using GridSearchCV. Based on the resulting density values, we kept 4,000 genes as high-density genes. These genes were then clustered using agglomerative hierarchical clustering with Euclidean distance and complete linkage. The optimal number of clusters (95) was selected by maximizing the silhouette score. From these, the biggest 30 clusters were kept for further analysis based on their expression profiles and enrichments.

### Quantification and statistical analysis

All analyses and visualizations presented in this manuscript (unless otherwise noted) were performed using PathwayFinder. For Figure 3, clustering of genes in the gene embedding was performed in Python using Scikit-learn, and the results were visualized using Matplotlib; detailed parameters are provided in method details. GO term enrichment analyses were performed using the g:Profiler API through the integrated interface in PathwayFinder. Reported enrichment *p* values were Bonferroni corrected; detailed settings are provided in method details.

## Supplemental information

Document S1. Figures S1–S3 and Table S1

Table S2. Gene modules sent for GO term analysis, related to Figure 3

Table S3. Gene modules sent for GO term analysis, related to Figure 4

## References

[1] Yao Z., van Velthoven C.T.J., Kunst M., Zhang M., McMillen D., Lee C., Jung W., Goldy J., Abdelhak A., Aitken M. (2023). A high-resolution transcriptomic and spatial atlas of cell types in the whole mouse brain. Nature.

[2] Van der Maaten L., Hinton G. (2008). Visualizing data using t-SNE. J. Mach. Learn. Res..

[3] McInnes L., Healy J., Melville J. (2018). Umap: Uniform manifold approximation and projection for dimension reduction. arXiv.

[4] Siletti K., Hodge R., Mossi Albiach A., Lee K.W., Ding S.-L., Hu L., Lönnerberg P., Bakken T., Casper T., Clark M. (2023). Transcriptomic diversity of cell types across the adult human brain. Science.

[5] Kobak D., Berens P. (2019). The art of using t-SNE for single-cell transcriptomics. Nat. Commun..

[6] Pezzotti N., Höllt T., Lelieveldt B., Eisemann E., Vilanova A. (2016). Hierarchical stochastic neighbor embedding. Comput. Graph. Forum.

[7] Van Unen V., Höllt T., Pezzotti N., Li N., Reinders M.J.T., Eisemann E., Koning F., Vilanova A., Lelieveldt B.P.F. (2017). Visual analysis of mass cytometry data by hierarchical stochastic neighbour embedding reveals rare cell types. Nat. Commun..

[8] Kolberg L., Raudvere U., Kuzmin I., Adler P., Vilo J., Peterson H. (2023). g: Profiler—interoperable web service for functional enrichment analysis and gene identifier mapping (2023 update). Nucleic Acids Res..

[9] Aleksander S.A., Balhoff J., Carbon S., Cherry J.M., Drabkin H.J., Ebert D., Feuermann M., Gaudet P., Harris N.L., Gene Ontology Consortium (2023). The gene ontology knowledgebase in 2023. Genetics.

[10] Zeng H. (2022). What is a cell type and how to define it?. Cell.

[11] Hao Y., Stuart T., Kowalski M.H., Choudhary S., Hoffman P., Hartman A., Srivastava A., Molla G., Madad S., Fernandez-Granda C., Satija R. (2024). Dictionary learning for integrative, multimodal and scalable single-cell analysis. Nat. Biotechnol..

[12] Wolf F.A., Angerer P., Theis F.J. (2018). SCANPY: large-scale single-cell gene expression data analysis. Genome Biol..

[13] Palla G., Spitzer H., Klein M., Fischer D., Schaar A.C., Kuemmerle L.B., Rybakov S., Ibarra I.L., Holmberg O., Virshup I. (2022). Squidpy: a scalable framework for spatial omics analysis. Nat. Methods.

[14] Megill C., Martin B., Weaver C., Bell S., Prins L., Badajoz S., McCandless B., Pisco A.O., Kinsella M., Griffin F. (2021). Cellxgene: a performant, scalable exploration platform for high dimensional sparse matrices. bioRxiv.

[15] Pielawski N., Andersson A., Avenel C., Behanova A., Chelebian E., Klemm A., Nysjö F., Solorzano L., Wählby C. (2023). TissUUmaps 3: Improvements in interactive visualization, exploration, and quality assessment of large-scale spatial omics data. Heliyon.

[16] Keller M.S., Gold I., McCallum C., Manz T., Kharchenko P.V., Gehlenborg N. (2025). Vitessce: integrative visualization of multimodal and spatially resolved single-cell data. Nat. Methods.

[17] Dries R., Zhu Q., Dong R., Eng C.-H.L., Li H., Liu K., Fu Y., Zhao T., Sarkar A., Bao F. (2021). Giotto: a toolbox for integrative analysis and visualization of spatial expression data. Genome Biol..

[18] Marconato L., Palla G., Yamauchi K.A., Virshup I., Heidari E., Treis T., Vierdag W.-M., Toth M., Stockhaus S., Shrestha R.B. (2025). SpatialData: an open and universal data framework for spatial omics. Nat. Methods.

[19] Huisman S.M.H., Van Lew B., Mahfouz A., Pezzotti N., Höllt T., Michielsen L., Vilanova A., Reinders M.J.T., Lelieveldt B.P.F. (2017). BrainScope: interactive visual exploration of the spatial and temporal human brain transcriptome. Nucleic Acids Res..

[20] Pezzotti N., Fekete J.D., Höllt T., Lelieveldt B.P.F., Eisemann E., Vilanova A. (2018). Multiscale visualization and exploration of large bipartite graphs. Comput. Graph. Forum.

[21] Zhang M., Pan X., Jung W., Halpern A.R., Eichhorn S.W., Lei Z., Cohen L., Smith K.A., Tasic B., Yao Z. (2023). Molecularly defined and spatially resolved cell atlas of the whole mouse brain. Nature.

[22] 10x Genomics (2024). Fresh Frozen Mouse Brain Hemisphere with 5K Mouse Pan Tissue and Pathways Panel. https://www.10xgenomics.com/datasets/xenium-prime-fresh-frozen-mouse-brain.

[23] Berkhout J.B., Poormoghadam D., Yi C., Kalsbeek A., Meijer O.C., Mahfouz A. (2024). An integrated single-cell RNA-seq atlas of the mouse hypothalamic paraventricular nucleus links transcriptomic and functional types. J. Neuroendocrinol..

[24] van Weert L.T.C.M., Buurstede J.C., Mahfouz A., Braakhuis P.S.M., Polman J.A.E., Sips H.C.M., Roozendaal B., Balog J., de Kloet E.R., Datson N.A., Meijer O.C. (2017). NeuroD factors discriminate mineralocorticoid from glucocorticoid receptor DNA binding in the male rat brain. Endocrinology.

[25] Wälchli T., Bisschop J., Carmeliet P., Zadeh G., Monnier P.P., De Bock K., Radovanovic I. (2023). Shaping the brain vasculature in development and disease in the single-cell era. Nat. Rev. Neurosci..

[26] Vieth A., Kroes T., Thijssen J., van Lew B., Eggermont J., Basu S., Eisemann E., Vilanova A., Höllt T., Lelieveldt B. (2024). ManiVault: A Flexible and Extensible Visual Analytics Framework for High-Dimensional Data. IEEE Trans. Vis. Comput. Graph..

[27] Wang Z., Dai R., Wang M., Lei L., Zhang Z., Han K., Wang Z., Guo Q. (2025). KanCell: dissecting cellular heterogeneity in biological tissues through integrated single-cell and spatial transcriptomics. J. Genet. Genomics.

[28] Pezzotti N., Thijssen J., Mordvintsev A., Höllt T., Van Lew B., Lelieveldt B.P.F., Eisemann E., Vilanova A. (2020). GPGPU linear complexity t-SNE optimization. IEEE Trans. Vis. Comput. Graph..

[29] Pedregosa F., Varoquaux G., Gramfort A., Michel V., Thirion B., Grisel O., Blondel M., Prettenhofer P., Weiss R., Dubourg V. (2011). Scikit-learn: Machine learning in Python. J. Mach. Learn. Res..

