# Supplementary material for: PathwayFinder enables linked exploration of cell types and gene modules in spatially resolved transcriptomics data
Source: iScience. 2026 Oct 29;29(11):118043. doi: 10.1016/j.isci.2026.118043 (PMC13742857; doi:10.1016/j.isci.2026.118043)
Supplement: Supplementary file 1 [file mmc1.pdf]

iScience, Volume *29*

Supplemental information

PathwayFinder enables linked exploration of cell types and gene modules in spatially resolved transcriptomics data

Chang Li, Julian Thijssen, Thomas Kroes, Lisa Koorneef, Thomas Höllt, Louise van der Weerd, and Boudewijn Lelieveldt

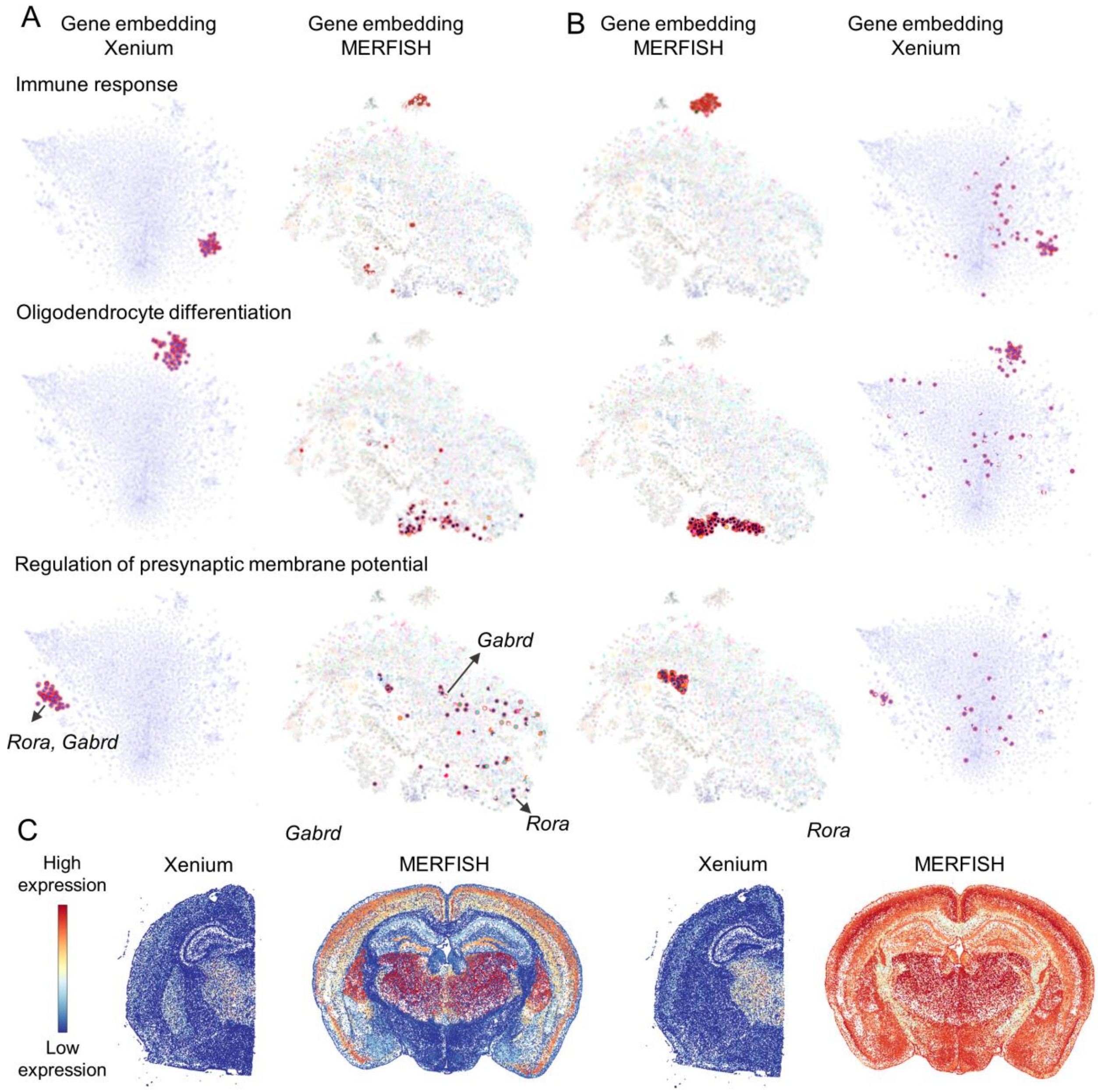

Supplemental Figure 1. Comparisons of gene embeddings from Xenium Prime mouse brain hemisphere[22] and Yao et al.[1] MERFISH mouse brain coronal section 38, related to Figure 2 and Figure 4. A. Selecting gene clusters in the Xenium gene embedding and highlighting the corresponding genes (if exist) in the MERFISH embedding. **B.** Selecting gene clusters in the MERFISH gene embedding and highlighting the corresponding genes (if exist) in the Xenium embedding. **C.** Genes that are not preserved in the same cluster show different expression patterns across datasets.

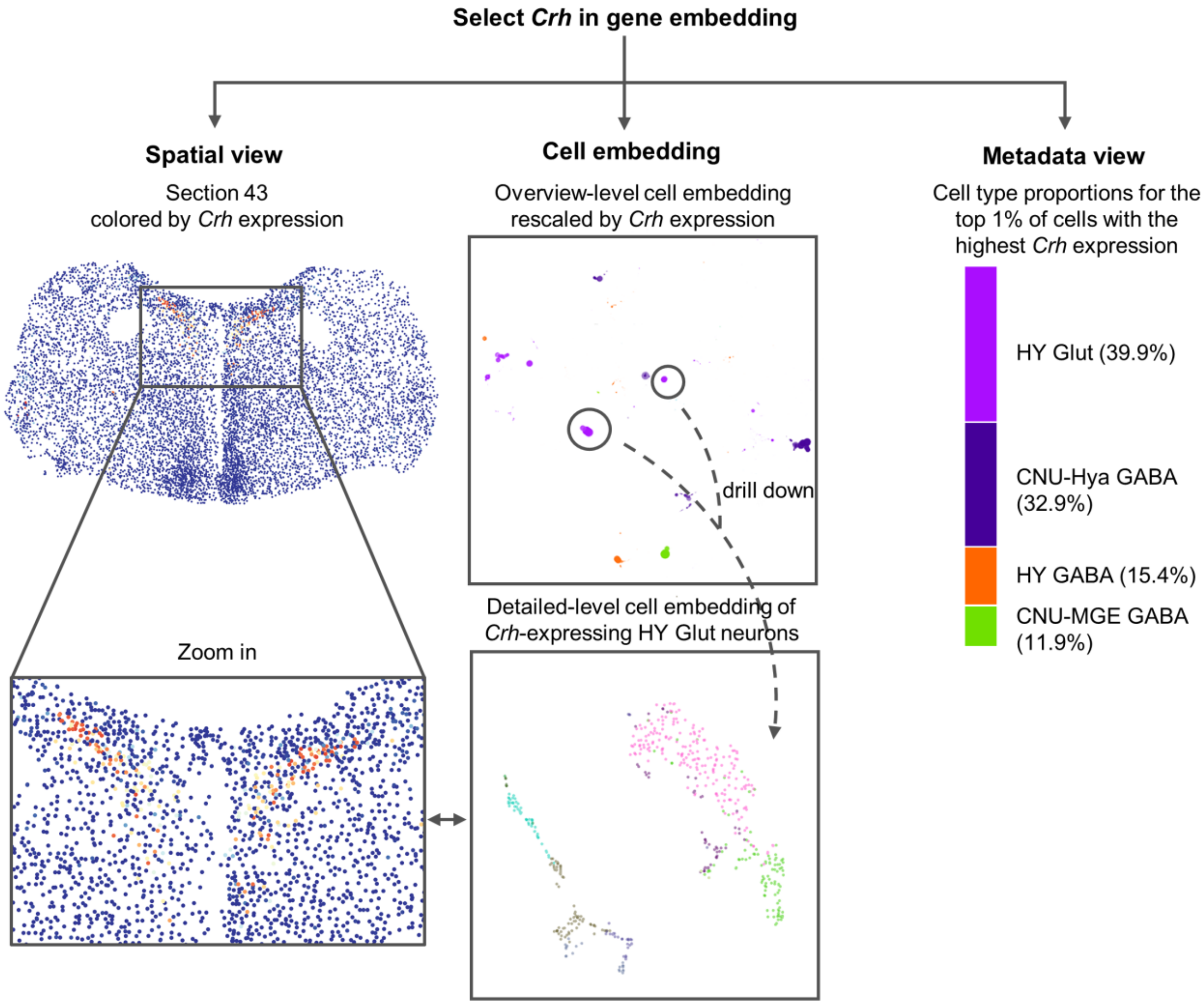

Supplemental Figure 2. Linked multi-view updating and hierarchical drill-down during the *Crh* exploration pipeline, related to Figure 5. Selecting the *Crh* gene simultaneously updates the spatial view, cell embedding, and metadata view. The spatial view is colored by *Crh* expression and can be interactively zoomed in to inspect highly expressing regions. The cell embedding illustrates the transition from a global overview-level embedding to a detailed embedding of the *Crh*-expressing HY Glut neurons. The metadata view provides a bar chart showing the proportional distribution of *Crh* expression across the cell types.

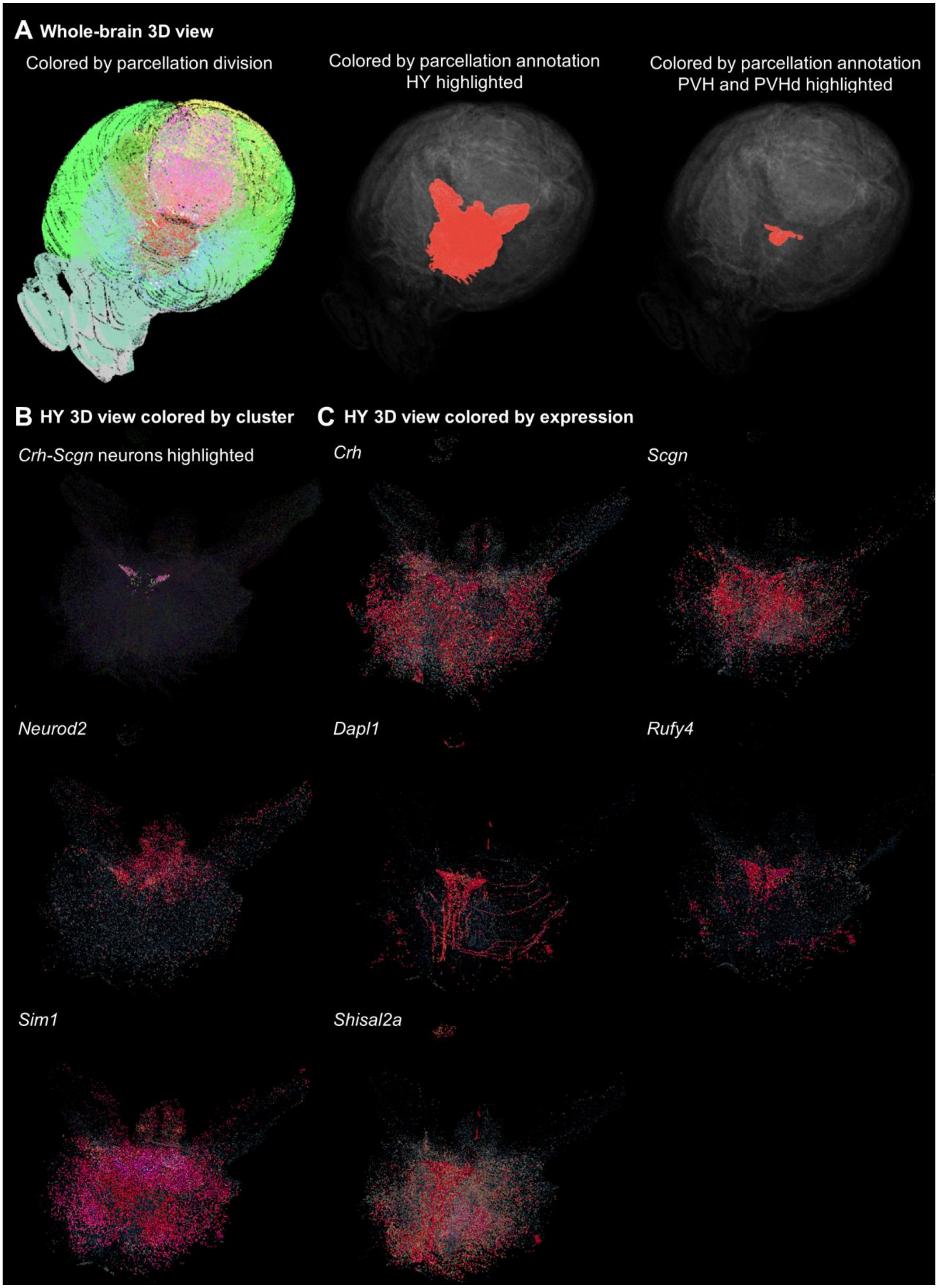
A Whole-brain 3D view
Colored by parcellation division
Colored by parcellation annotation
HY highlighted
Colored by parcellation annotation
PVH and PVHd highlighted
B HY 3D view colored by cluster
Crh-Scgn neurons highlighted
C HY 3D view colored by expression
Crh
Scgn
Neurod2
Dapl1
Rufy4
Sim1
Shisal2a

Supplemental Figure 3. Visualization of 3D representations of Yao et al.[1] MERFISH mouse brain, related to Figure 5. A. Whole-brain 3D view colored by parcellation annotations. Left: whole-brain visualization colored by "parcellation division". Middle: whole-brain shown in the background, with hypothalamic structures highlighted and colored by "parcellation structure". Right: whole-brain shown in the background with paraventricular hypothalamic nucleus (PVH) and the descending division of the PVH. (PVHd) structures highlighted and colored by "parcellation structure" **B.** 3D view of the hypothalamus with *Crh-Scgn* neurons highlighted and colored by "cluster". **C.** 3D view of the hypothalamus colored by gene expression,where both color intensity (red) and opacity encode expression level. Higher expression appears more intense and more opaque.

Supplemental Table 1. Data size, dimension reduction parameters, memory usage and project loading times for the datasets analyzed in this paper, related to Methods.

	MERFISH mouse brain imputed coronal section 38 from Yao et al.[1]	MERFISH mouse brain imputed hypothalamus from Yao et al.[1]	MERFISH mouse brain imputed ABCA-4 from Zhang et al.[21]	Xenium Prime mouse brain hemisphere dataset[22]
Data dimension [cells, genes]	[120186, 8460]	[132902, 8460]	[162578, 20000]	[63173, 5006]
Cell embedding parameters	HSNE: 2 layers, random seed 42, otherwise default	HSNE: 2 layers, random seed 42, otherwise default	HSNE: 2 layers, random seed 42, otherwise default	t-SNE: random seed 42, otherwise default
Gene embedding parameters	t-SNE: default settings	t-SNE: default settings	t-SNE: default settings	t-SNE: default settings
Memory usage	8.5GB	9.31GB	25.39GB	2.90GB
Project loading time on workstation	33.87s	37.40s	81.15s	10.19s
